# Naturally present metal ions in plants could interfere with common antioxidant assays

**DOI:** 10.1016/j.mex.2020.100995

**Published:** 2020-07-16

**Authors:** Teodora Tomova, Venelin Petkov, Iva Slavova, Plamen Stoyanov, Mariana Argirova

**Affiliations:** a Department of Chemical Sciences, Faculty of Pharmacy, Medical University of Plovdiv, 15^a^ Vassil Aprilov Blvd, 4002 Plovdiv, Bulgaria; b Department of Botany and Methods of Biology Teaching, Faculty of Biology, University of Plovdiv “Paisii Hilendarski”, 24 Tzar Assen Str., 4000 Plovdiv, Bulgaria; c Department of Bioorganic Chemistry, Faculty of Pharmacy, Medical University of Plovdiv, 15ª Vassil Aprilov Blvd, 4002 Plovdiv, Bulgaria

**Keywords:** Antioxidants, Total polyphenol content, DPPH, Ferric reducing capacity, Ferrozine, Metal chelates

## Abstract

Most of the commonly applied assays used to assess antioxidant properties of plant extracts exploit the ability of some biologically active metabolites to participate in oxidation-reduction reactions with metal ions. On the other hand, most plants contain different chelated metal ions whose levels depend on the geographic origin, soil, and environmental pollutions. In this study the levels of redox-active metal ions in three plant sources were measured and extracts of these botanicals were treated with Chelex^Ⓡ^ – an ion exchanger that is noteworthy for its ability to bind transition metal ions. The original and chelated extracts were subjected to three antioxidant assays based on single electron transfer. The results obtained showed statistically significant differences between the original and Chelex-treated extracts suggesting that the naturally present metal ions could interfere with the results of the three most commonly applied antioxidant methods.

• The proposed pre-analytical procedure is simple and does not require special instrumental equipment.
• Preliminary depletion of redox active metal ions, namely iron and copper ions could improve reproducibility of the analytical methods.
• The method allows a more reliable comparison of antioxidant properties of particular botanical species from different geographic regions.

## Graphical abstract

**Figure:**
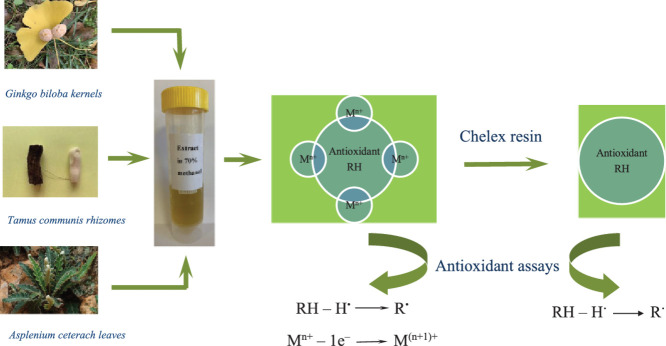

Specifications table

**Table:** 

Subject Area	Agricultural and Biological Sciences
More specific subject area	*Analysis of natural products, foods and beverages*
Method name	*Simple approach to increase reproducibility and comparability of antioxidant methodology*
Name and reference of original method	*Not applicable*
Resource availability	*Not applicable*

## Method details

### Background

Oxidative stress is a pathological condition characterized by disturbed balance between production of reactive oxygen spices and antioxidant defense system in human organism and implicated in the onset and development of a number of diseases [1]. In recent years, the demand for new antioxidants capable of compensating the deficit of endogenous antioxidants or to counteract the increased production of reactive oxygen species is in focus of interest of numerous studies. Plants have been the basis of traditional folk medicine for thousands of years and continue to provide new remedies for the prevention and cure of different human diseases [2]. The plant kingdom is also practically unlimited source of low toxic antioxidants. These natural antioxidants can be taken up through diet as they are present in fruits, vegetables and spices.

The use of natural antioxidants is not limited to their beneficial effects for human health. Biodiesel, an alternative fuel derived from vegetable oils or animals fats is quite susceptible to oxidation and this process affects fuel quality. The use of synthetic antioxidants such as butylated hydroxytoluene delays the onset of oxidation but naturally sourced antioxidants can be just as effective as synthetic ones at the same time being inexpensive, non-toxic and environmentally friendly compared to their synthetic counterparts [3].

Several methods to evaluate the antioxidant activity of botanicals have been developed and published in the past decades, and the discussion about the real meaning and the usefulness of this kind of measurement is still open [4]; more over not all of them are relevant to human biology [5]. Recently published papers have tried to outline the chemistry, advantages and drawbacks of several methods and more or less to set some standards in the antioxidant methodology [6], [7], [8].

Most of the commonly applied assays used to assess antioxidant properties of plant extracts exploit either the ability of some biologically active plant metabolites, namely the broad group of polyphenols, to form colored chelates, or to participate in oxidation-reduction reactions with metal ions [9]. On the other hand, transition metal ions such as Fe, Cu, Ni, Mn and Zn are intrinsic components of enzymes and therefore essential for plant growth and survival. However, because at physiological pH these ions tend to form insoluble hydroxides, in living organisms they are chelated and kept in soluble bioavailable form as they are transported to cells and move through cellular compartments. The levels of these chelated metal ions very often depend on the geographic origin, soil, several environmental factors, pollutions and probably many other variables. We hypothesized that these naturally present chelated and redox-active metal ions in plants could interfere with the results of antioxidant assays and thus would not allow a correct comparison of antioxidant properties of plant extracts even from one and the same botanical species. We tested this hypothesis on three plant extracts treated with Chelex^Ⓡ^ 100 ion exchange resin - a styrene divinylbenzene copolymer containing paired iminodiacetate ions. This resin has a strong selectivity for copper, iron, and other heavy metal ions and effectively remove them from buffers [10]. Three common antioxidant methods based on single electron transfer were applied. This mechanism is applicable to the redox cycling Fe(III)/Fe(II) and/or Cu(II)/Cu(I) that might operate in the presence of these metal ions in plant extracts.

## Materials and Methods

### Plant extracts

Five grams of plant material (*Ginkgo biloba* kernels, *Tamus communis* rhizomes, and *Asplenium ceterach* leaves) were extracted with 50 mL 70% methanol by stirring at room temperature for 8 h in a light protected place. The extract was filtered with Whatman No. 1 filter paper and the extraction was repeated two more times. The combined extracts were stored at 4 °C prior to the analyses. Dry matter content of these extracts was determined gravimetrically; the results were average of two parallel samples.

Commercial Chelex^Ⓡ^ resin was preliminary washed by stirring overnight with 70% methanol and dried on air. Five grams of this conditioned resin was stirred with 25 mL plant extract overnight at room temperature. A simple test based on ascorbic acid autoxidation was applied to prove the absence of free or loosely bound redox active metal ions in the extracts [11]. Afterward the extract was filtered and its dry matter was determined gravimetrically in duplicate.

### Antioxidant assays

Total phenolic content of the extracts was determined using protocol 01/2008:20814 of the European Pharmacopeia with minor modifications [12]. The method proposed by Berker et al. [13] was used to measure the total antioxidant capacity of plant extracts. Experimental conditions of the radical-scavenging propertied assay were set according to the method proposed by Serpen et al. [14]. For details see Supplementary material.

## Results and discussion

The content of three microelements in plants of interest measured by ICP-MS (for details see Supplementary material) is shown in Table 1. Of these, particular interest is the concentration of the redox active iron and copper. While the levels of copper in the three plants were quite similar and in the same order of magnitude, the content of iron ions in the leaves of *Asplenium ceterach* was very high. This fern species is commonly known as *Rustyback* and the specific color of its leaves may be due to the high content of iron compounds.

**Table 1:** Concentrations (in ppm in relation to dry specimens) of three metals in the studied plants. Values are mean of four scans ± standard deviation.

Botanical species	Iron (ppm)	Copper (ppm)	Zinc (ppm)
*Ginkgo biloba* kernels	26.0 ± 1.4	6.8 ± 0.4	17.2 ± 0.8
*Tamus communis* rhizomes	20.3 ± 2.1	5.3 ± 0.5	76.5 ± 6.9
*Asplenium ceterach* leaves	151.3 ± 4.5	6.4 ± 0.3	76.0 ± 3.3

**Fig. 1:**
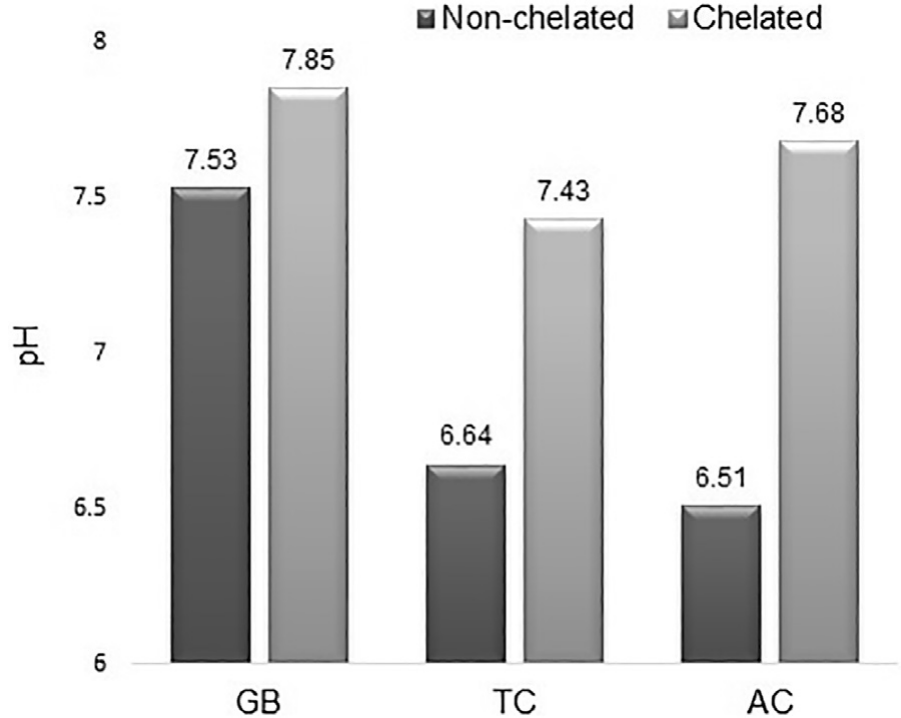
Changes in pH values of the extracts with concentration 1 mg/ml before and after treatment with Chelex resin. GB – *Ginkgo biloba* kernels, TC – *Tamus communis* rhizomes, AC – *Asplenium ceterach* leaves.

Since pH value is meaningful for water solutions, both the chelated and non-chelated 70% methanolic extracts were diluted with water to final concentration 1 mg/ml dry matter. pH values of these solutions at 25.0°C are shown in Fig. 1. The pH shift of chelated solution toward higher values suggests replacement of coordinately bound to phenol groups transition metal ions with ionic bound sodium from Chelex resin. This change in pH of the tested solutions is however unlikely to interfere with two of the antioxidant assays because total polyphenol content is measured in basic medium (pH ~10) and DPPH assay is performed in methanol. Moreover, these assays use small volumes of plant extracts compared to the total volume of analyzed solution. The only pH sensitive method is the assay of total antioxidant capacity but it uses large volume of acetate buffer with sufficient buffer capacity.

The Folin-Ciocalteu reagent contains phosphomolybdic/ phosphotungstic acid complexes. The quantification of total polyphenol content relies on the transfer of electrons in alkaline medium from phenolic compounds to the reagent that is reduced and forms a blue chromophore constituted by a phosphotungstic/phosphomolybdenum complex. Molibdates are more easily reduced than tungstates and most likely phenols donate an electron for the transition Mo (VI) → Mo (V). Good correlation between total polyphenol content and antioxidant activity of plant extracts measured by other methods has been reported in numerous studies [15,16].

Upon the basic pH of the assay phenolic compounds are almost completely dissociated to phenoxide ions that are the real reductants. This mechanism was named Sequential Proton-Loss Electron Transfer (SPLET) [17]:

(1) $$\text{ArOH}\to \text{Ar}O^{-}$$

(2) $$\text{Ar}O^{-}\to \text{Ar}O^{.}+e^{-}$$

Single electron delocalization of phenoxide radical in those of polyphenols with ortho or para hydroxyl groups makes this reaction thermodynamically favorable and the free radical formed may further react with a second radical; a reaction that turns the phenolic group into a stable quinone structure. Both the configuration and the total number of hydroxyl groups substantially influence the antioxidant activity of polyphenols.

Our expectation was that after the release of the phenolic groups from their coordination compounds with transition metal ions, the total polyphenolic content of the extracts would increase. Data shown in Table 2 revealed such tendency only for the extract obtained from *Ginkgo biloba* kernels while the other two botanical chelated extracts had lower total polyphenol content. Apparently, in the original plant extracts there had been another reductant that had been removed after Chelex treatment; most likely that was Fe(II). In fact, it has been shown that at physiological relevant pH (6.8 and 7.5) 24 of totally 26 tested flavonoids have been potent Fe(II) chelators [18].

**Table 2:** Antioxidant activities of methanolic extracts obtained from *Ginkgo biloba* kernels, *Tamus communis* rhizomes and *Asplenium ceterach* leaves and of the same extracts after treatment with Chelex^Ⓡ^ ion exchange resin in sodium form measured by three methods. Values are means of triplicate measurements ± standard deviation. Statistically significant difference between the values obtained for one and the same plant source was evaluated by the P-value for each comparison.

Botanical species	Total polyphenols (GAE)		Total antioxidant capacity (Trolox eq)		Radical scavenging properties (µg IC50)	
	Mean ± SD	P	Mean ± SD	P	Mean ± SD	P
*Ginkgo biloba* (non-chelated)	118 ± 3	0.005	98 ± 5	0.006	470 ± 10	0.0001
*Ginkgo biloba* (chelated)	128 ± 2		115 ± 1		620 ± 20	
*Tamus communis* (non-chelated)	112 ± 2	0.04	336 ± 4	0.0001	720 ± 70	0.04
*Tamus communis* (chelated)	104 ± 4		309 ± 4		940 ± 100	
*Asplenium ceterach* (non-chelated)	2108 ± 60	0.0002	15974 ± 32	0.00001	35 ± 0.4	0.0003
*Asplenium ceterach* (chelated)	1649 ± 9		10947 ± 63		53 ± 1.4	

Theoretically the good antioxidant must be a good reductant and quantification of the reducing power is widely used to evaluate the antioxidant activity of plant extracts rich in biologically active compounds which possess potent electron-donating abilities. Similarly to the total polyphenol assay, this method uses an electron transfer from a reductant to Fe(III) which is reduced to Fe(II). The latter is quantified by measuring the formation of magenta-colored coordination compound with Ferrozine.

If we assume that under the reductive environment of the plant cells iron and copper ions are mostly in their reduced form and bound to polyphenols, that could explain the higher reducing capacity of non-treated plant extract that was diminished after removal of transition metal ions with Chelex (Table 2).

DPPH (2,2-diphenyl-2-picrylhydrazyl) free radical assay is based on the reduction of stable DPPH nitrogen radical in the presence of antioxidants. This assay was carried out in methanol solution in which apart from single electron transfer another mechanism: hydrogen transfer, is in operation. According to Litwinienko and Ingold [17] the reaction of phenols with DPPH in alcoholic solvents is due to the presence of traces of phenoxide ions that react rapidly with electron-deficient DPPH radical according to the equation:

(3) $$\text{Ar}O^{-}+\text{DPP}H^{.}\to \text{Ar}O^{.}+\text{DPP}H^{-}$$

(4) $$\text{DPP}H^{-}+H^{+}\to \text{DPPH}-H$$

Considering Henderson – Hasselbalch equation it can be calculated that the degree of ionization of phenol group at pH 7.4 is around 28% and methanol supports the ionization.

Another scenario that may be considered in this assay of plant extracts is a fast transfer of one electron from chelated metal ions in their low oxidation state to DPPH radical followed by a transfer of proton from ionizable phenol groups:

(5) $$M^{n+}-e^{-}\to M^{\left(n+1\right)+}$$

(6) $$\text{DPPH}+e^{-}\to \text{DPP}H^{-}$$

(7) $$\text{DPP}H^{-}+H^{+}\to \text{DPPH}-H$$

All native plant extracts were better radical scavengers than Chelex treated ones and this finding supports the above assumption.

There were several unknowns at the beginning of this study: i) Whether chelated iron and copper ions are soluble in 70% methanol and present in the extracts; ii) Whether these chelated metal ions are still redox active, and iii) The elemental analysis provides information about the total pool of metal ions but not information about their redox state; an information that is essential for their biological functions. This study answered some of the questions: apparently metal chelates were extractable in 70% methanol as evidenced by the changes in color, UV-VIS spectra and pH of the extracts after depletion of chelated ions with Chelex resin. These chelated ions were redox active because their presence or absence in the extracts gave different results in the assays based on single electron transfer. Regarding their redox state, the results obtained for the antioxidant activity of the native and chelated extracts indicate that the Fe (II) and/or Cu (I) most likely to prevail, but it is not clear whether these are the original ions present in the plant sources or they were obtained during the isolation of herbal antioxidants. One of the most widely spread flavonoids, quercetin, undergoes significant autoxidation at pH 7.4 that may be attributed to metal traces but this autoxidation is strongly accelerated by addition of metal ions [19]. A redox shuttle between two oxidation states of chelated metal ions in the complex plant extracts can also be considered in the light of the finding that phenolic compounds under certain conditions can act as pro-oxidants [20].

## Conclusions and future prospects

To the best of our knowledge this is the first attempt to shed some light on the role of naturally present metal ions on antioxidant properties of plant extracts and the results obtained unequivocally show that these ions even in ppm levels can interfere with some of the commonly applied antioxidant methods. In fact, the levels of active constituents present even in one and the same medicinal plant show remarkable variability due to location, seasonal, climatic and agronomic factors, post-harvesting handling, etc. The content of metal ions in plant is also highly variable but most often associated with soil pollution. Preliminary depletion of metal ions in the plant extracts, namely iron and copper ions is a simple procedure that could improve reproducibility of the antioxidant methods and allows a more reliable comparison of antioxidant properties of particular botanical species from different origin. This procedure applied to natural antioxidants used to increase biodiesel stability would improve their effectiveness in preventing lipid peroxidation of unsaturated fatty acids, a process induced by traces of redox active metal ions [21].

## CRediT authorship contribution statement

**Teodora Tomova:** Investigation, Writing - original draft. **Venelin Petkov:** Investigation, Formal analysis. **Iva Slavova:** Investigation, Formal analysis. **Plamen Stoyanov:** Resources. **Mariana Argirova:** Conceptualization, Methodology, Writing - review & editing.

## Appendix Supplementary materials

Image, application 1
