# Supplementary material for: Naturally present metal ions in plants could interfere with common antioxidant assays
Source: MethodsX. 2020 Jul 16;7:100995. doi: 10.1016/j.mex.2020.100995 (PMC7390811; doi:10.1016/j.mex.2020.100995)
Supplement: Supplementary file 1 [file mmc1.docx]

**Plant material**

According to the Bulgarian floristic zoning, the area of plant material collecting is in Floristic Region No 18.

*Ginkgo Biloba* fruits were collected in November 2018 from a tree on the yard of Medical College in Plovdiv, Bulgaria. They were shelled, crushed manually in a glass mortar with a pestle and stored in plastic packages at -20 ^o^C until use.

Rhizomes of *Tamus Communis* were collected in April 2019 in an ecologically clean area near the town of Asenovgrad, Plovdiv district. The rhizomes were washed thoroughly under tap water to make free from contaminants. Inner cork skin was peeled off using ceramic knife and the white core of rhizomes was cut in small pieces, smashed in a glass mortar and stored at –20 ^o^C prior to the assays.

Plant material from *Asplenium ceterach* was collected in the fall of 2019 at the Asen Fortress, Asenovgrad, Plovdiv district. The leaves were dried at ambient temperature of 23–25 °C in dark in order to avoid degradation of light sensitive pigments and polyphenolic compounds. The dried herb was crushed, packed in paper bags and stored at ambient temperature prior to the extraction and analysis.

All the plants used were identified by Prof. Plamen Stoyanov from the Department of Botany and Methods of Biology Teaching, Faculty of Biology, University of Plovdiv “Paisii Hilendarski”.

**Determination of trace elements in plant material by ICP-MS**

A microwave digestion procedure was performed in order to obtain complete sample dissolution. Plant materials were dried at 120 ^o^C to constant weight. After grinding in a glass mortar 0.100 g of sample was mineralized with 2 mL of conc. HNO_3_ and 1 mL deionized water at 190 °С. The following oven program (Multiwave Go, Anton-Paar) was used: ramp 20 min., hold 20 min, cooling 10 min. After mineralization the samples are brought up to 30 ml with deionized water and analyzed by inductively coupled plasma mass spectrometry (ICP-MS) (Thermo Fisher Scientific, iCAPQ) in KED mode using 103Rh as an internal standard. Reference material NCS DC 73348 was analyzed in parallel for evaluating the accuracy of the digestion process. Multi-element acid solutions were used for the calibration, thus providing an accurate quantitative analysis.

**Antioxidant methods in brief**

***Total polyphenols content determination***

Each methanolic extract (0.1 ml) was mixed with 0.5 ml of commercial *Folin–Ciocalteu* reagent diluted 1:10 (v/v); 4 minutes later 0.4 ml water solution of Na_2_CO_3_ (7.5%, w/v) was added. After 30 min the absorbance at 765 nm was recorded. Standard solutions of gallic acid in 70% methanol (concentration range 20-100 μg/mL) were used for calibration line. Results were expressed as mg gallic acid equivalent (GAE) per gram dry extract, i.e. mg GAE having the same optical density at 765 nm as 1 g dry extract. Each value represents the mean ± SD (n = 3).

***Total reducing capacity assay***

Three hundred μl Fe-Ferrozine reagent (2 mM FeCl_3_.6 H_2_O and 10 mM Ferrozine) was mixed in plastic cuvette with 400 μl acetate buffer (200 mM, pH 5.5) and 100 μl 70 % methanolic extract was added. The absorbance at 562 nm was measured 30 min latter. Solutions of Trolox in 70% methanol in the concentration range 20-100 μM were used for calibration plot and the results for antioxidant capacity were expressed as Trolox equivalents, i.e. millimoles Trolox causing the same reduction of the Ferric-Ferrozine complex as 1 g dry extract). Each determination of the antioxidant capacity was conducted in triplicate, and the results were expressed as mean values ± standard deviation.

***Radical-scavenging properties***

Aliquots (100 µL) of extracts with various concentrations (0-1 mg/ml) were added to 1.7 mL of a 60 μM solution of DPPH in methanol in order to obtain calibration curves and to calculate IC_50_ values. The mixture was thoroughly vortexed, kept in the dark for 30 min, and the absorbance at 517 nm was measured using methanol as a blank. Antiradical activity was defined as the dry extract in μg providing 50% inhibition (IC_50_) of the initial DPPH and was calculated from the graph plotting absorbance at 517 nm against extract concentration. For reasons of clarity, the lower IC_50_, the more efficient the antioxidant. Three replicates were used and the average absorption was calculated for each concentration point.

Data were processed using Microsoft® Office Excel. Student’s t-test was used for statistical analyses; P values > 0.05 were considered to be significant.
